# Social interactions and olfactory cues are required for contagious itch in mice

**DOI:** 10.1038/s41598-024-61078-3

**Published:** 2024-05-17

**Authors:** Maryam Shayan, Nazgol-Sadat Haddadi, Maryam Shokrian Zeini, Mohadese Shokrian Zeini, Hasti Tashak Golroudbari, Arya Afrooghe, Elham Ahmadi, Asma Rashki, Ahmad-Reza Dehpour

**Affiliations:** 1 Experimental Medicine Research Center, Tehran University of Medical Sciences, Tehran, Iran; 2 Department of Ophthalmology, Harvard Medical School, Schepens Eye Research Institute of Massachusetts Eye and Ear, Boston, MA USA; 3 Department of Dermatology, University of Massachusetts Chan Medical School, Worcester, MA USA; 4 Department of Pharmacology, School of Medicine, Tehran University of Medical Sciences, Poorsina St., Enghelab Ave., P.O. Box 13145-784, Tehran, Iran; 5 Department of Internal Medicine, University of Michigan, Ann Arbor, MI USA

**Keywords:** Contagious itch, Emotional contagion, Olfactory system, Olfaction, Social behaviour, Olfactory system

## Abstract

The phenomenon of contagious itch, observed in both humans and rodents, remains a topic of ongoing debate concerning its modulators and underlying pathways. This study delves into the relationship between contagious itch and familiar olfactory cues, a non-visual factor contributing to this intriguing behavior. Our findings showed that contagious itch in observer mice occurs during physical interaction with the cagemate itch-demonstrator but not with a stranger demonstrator or in a non-physical encounter condition. Notably, itch-experienced observer mice displayed an increased contagious itch behavior, highlighting the relevance of itch-associated memory in this phenomenon. Furthermore, anosmic observer mice, whether itch-naïve or itch-experienced, displayed no contagious itch behavior. These results demonstrate that the familiar olfactory cues, specifically cagemate body odors, are required for contagious itch behaviors in mice. In line with these behavioral findings, our study reveals increased activity in brain regions associated with olfaction, emotion, and memory during contagious itch, including the olfactory bulb, the amygdala, the hypothalamus, and the hippocampus, with this activity diminished in anosmic mice. In conclusion, our study unveils the critical role of familiar olfactory cues in driving contagious itch in mice, shedding light on the interplay between social factors, sensory perception, and memory in this phenomenon.

## Introduction

Itch is an unpleasant sensation that typically prompts a scratching response^1^. Pruritogens stimulate cutaneous pruriceptors, which then transmit itch signals via the glutamatergic projection of afferent C-fibers to the spinal dorsal horn. The thalamus receives this information from the pruriceptors, and itch-related cortical centers, such as the prefrontal cortex, the somatosensory cortex, the premotor areas, the insular cortex, and the anterior cingulate cortex (ACC), process the information^1^.

Contagious itch is a phenomenon that has been observed in humans and other primates^2–5^. Audiovisual stimuli can provoke an itch in humans, while others may display scratching behavior or feel an itch when discussing scratch-related topics. Viewing a scratching video or listening to a lecture on a scratch-related subject can also trigger an itch sensation in humans^6^. Contagious itch has also been reported in monkeys placed with a scratching cagemate or shown videos of monkeys scratching^4^.

In recent years, studies have investigated whether contagious itch is present in rodent models. Yu et al.^7^ have shown that imitative scratching behavior occurs in naïve mice, even without prior itch experience or a promising reward, while observing conspecific scratching videos or actual genetically modified mice with excessive scratching. Gonzales-Rojas et al.^8^ also showed that imitative video-induced scratching is present in FVB mice using a similar method developed by Yu et al^7^.

Lloyd et al.^2^ have suggested that empathy plays a role in contagious itch, as human participants reported a high itch sensation for themselves and the person in a static image experiencing an itch. However, it is still unclear whether contagious itching observed in animal models is a form of motor mimicry, emotional contagion, or empathy. The specific itch-related stimuli in animal models have yet to be fully understood, and it is uncertain whether contagious itch induced by watching an itching video is reproducible in rodents.

Yu et al.^7^ showed that contagious itch in mice is due to visual inputs, with increased neuronal activity in the gastrin-releasing peptide receptor (GRPR) neurons of the suprachiasmatic nucleus (SCN) of mice with contagious scratching. Gao et al.^9^ expanded on this study and revealed that the visual cortex and superior colliculus are not involved in contagious itch. Instead, a novel pathway involving intrinsically photosensitive retinal ganglion cells (ipRGCs)-SCN-paraventricular thalamic nucleus (PVT) transmits the signals essential for contagious itch behavior.

In this study, we aim to investigate whether olfactory or auditory cues are involved in contagious scratching/itching in mice. Additionally, we will explore the possible involvement of the central olfactory and associated pathways, as well as experience-dependent potentiation, in developing contagious itch-related behavior. Ultimately, our study aims to shed light on whether contagious itch in animal models can be attributed to empathic behaviors.

## Materials and methods

### Chemicals

Compound 48/80 (PubChem CID: 104735) and Kanamycin sulfate (PubChem CID: 32943) were purchased from Sigma Chemicals (U.K). Compound 48/80 (C48/80) was prepared freshly for use by liquefying in 50 µl of physiological saline and administered at 100 µg per site. Kanamycin sulfate was dissolved in physiological saline and administered at a dose of 1000 mg/kg via the intraperitoneal route. Furosemide (PubChem CID: 3440) was purchased from Centrafarm, Netherlands. Furosemide was administered intraperitoneally at a dose of 100 mg/kg. Zinc sulfate (ZnSO_4_) (PubChem CID: 24424) was purchased from Merck Chemicals, Germany. The 5% zinc sulfate solution was prepared by dissolving the chemical in physiological saline, and 50 µl of the 5% solution was injected into each nostril using a 100 µl micropipette. Lidocaine hydrochloride (PubChem CID: 6314) with a 2% concentration in a sterile injection solution was purchased from Shahid Ghazi Pharmaceutical CO, Tabriz, Iran. For local anesthesia, 2 or 3 drops of lidocaine were injected into each nostril using a 100 µl micropipette.

### Animals

A total of 272 male NMRI mice (Pasteur Institute, Tehran, Iran) aged 5–6 weeks and weighing 25–30 g were included in the study. All the mice were maintained in a controlled environment in terms of temperature (23–25 °C) and lighting (lights on from 08:00 AM to 08:00 PM). Access to food and water was unrestricted. All procedures were performed following the institutional guidelines for animal care and use (NIH Publications No. 8023, revised) and also ARRIVE guidelines for animal research. All the operational guidelines in the housing, routine husbandry, handling, and experimental procedures were approved by the committee for animal ethics and experiments at Tehran University of Medical Sciences, Tehran, Iran. (Approval number: 99-1-101-47031). All the mice were used once for each experiment.

### Behavioral experiments

Before the experiment, the mice were carefully checked for any signs of dermatological disorders. To acclimate them to the environment, each mouse was placed in a separate acrylic box (10 × 10 × 13 cm) with a small amount of bedding for 30 min daily for three days prior to the behavioral experiment. On the day of the experiment, two mice (one demonstrator mouse and one observer mouse) were placed together in the same box. Compound 48/80 (100 µg/site) was then administered intradermally in the nape of the neck of the demonstrator mice to induce itching.

The experiment was recorded for one hour using a digital camera mounted on a camera stand to minimize distraction and maximize stabilization. The videos were later analyzed to estimate the number of scratching behaviors. For the mice injected with C48/80, the total number of scratching bouts was quantified as the lifting of the hind paw to scratch the injection site and then returning the hind paw to the ground or the mouth. For the observer mice, three parameters were counted: (1) the total number of looks towards the demonstrator, (2) the total number of looks and scratching bouts within 5 s of each other (look-scratch (all look-and-scratch should be look-scratch instead) (Supplementary Video S7), and (3) the total number of scratching bouts, which were defined as lifting the hind paw to scratch the face, neck, back, or sides and then returning the hind paw to the ground or the mouth. A look towards the demonstrator mice was defined as a stop-and-look behavior exhibited by the observer mice. All animal handling, drug injections, behavioral experiments, and counting of the number of scratches were carried out by a skilled investigator. The entire visual field of the mice was considered while counting the number of looks^10^.

All the mice used in the experiment were of the same sex, age, strain, and home cage and were kept in the same environment with free access to the same food and water. The experiments were conducted between 8 and 10 AM.

### Experimental groups

#### Contagious itch in mice

To examine the imitation of itching, observer mice were randomly assigned to one of two groups. The first group, Physical Encounter (PE), consisted of mice that had physical contact with the scratching demonstrator mice during the experiment. The second group, Non-Physical Encounter (NPE), consisted of mice that were separated from the scratching demonstrator mice with a perforated transparent plastic layer to prevent physical contact but allow for visual, olfactory, and auditory exposure. On the day of the experiment, observer and demonstrator mice were placed in their respective boxes according to their assigned group. The itch demonstrator mouse was briefly removed from its box for intradermal injection of C48/80 (100 µg/site) and then returned to the same box after the injection. The experiment was recorded for one hour, and the behavior of the observer mouse was analyzed. The number of mice used in each group was as follows: (1) PE group: observer mice (n = 8) next to the scratching demonstrator mice (n = 8) who received the injection; (2) NPE group: observer mice (n = 8) physically separated from the scratching demonstrator mice (n = 8) who received the injection. In the control group, observer mice witnessed saline-injected mice as the itch demonstrator. An overview of the key components of the experimental setup and the flow of the study can be found in Supplementary Fig. S1.

#### The role of familiarity in contagious itch

The study included two groups of mice, familiar and unfamiliar, each consisting of 16 mice (8 observers and 8 demonstrators). In the familiar group, the mice were housed in the same home cage for at least two weeks. Each paired mouse from the same family cage was habituated in one test box for 30 min daily for three days before the behavioral experiment. On the day of the experiment, the mice were placed in their respective boxes next to each other, and the itch demonstrator mice were injected with C48/80 (100 µg/site). In the unfamiliar group, the observer and demonstrator mice were selected from different family cages and were habituated separately in different boxes before the experiment. The observer mice were habituated with their cagemates in their respective boxes for three days. On the day of the experiment, the observer mice were introduced to the unfamiliar itch demonstrator mice for the first time in the box where they had been habituated, and the demonstrator mice were injected with C48/80 (100 µg/site). The behaviors of the observer mice towards the unfamiliar itch demonstrator mice were recorded and analyzed.

Prior studies have documented the effect of foot shocks to induce acute stress. Further previously, it has been demonstrated that there could be a link between acute stress and pruritus sensation^11–13^. Hence, to resemble the acute stress of a stranger mouse and explore the effect of such stress on contagious itch, we conducted a foot shock test before a contagious itch. In this experiment, the observer mice were subjected to foot shocks for 30 min one hour before the test. During the 30 min, the mice received 60 electric shocks with a strength of 0.4 mA and a duration of 1 s, with random inter-trial intervals of 15–45 s.

#### The role of smell and hearing senses in contagious itch

To induce anosmia in the observer mice, zinc sulfate was administered intranasally after anesthesia with lidocaine hydrochloride. The food-finding test (FFT) was performed one day before the experiment to confirm anosmia in the observer mice. In this test, food-deprived mice were placed in a clean cage with a chew hidden 0.5 cm beneath the bedding. Mice were considered anosmic if they could not find the chew within 2 min^14^. On the experiment day, the anosmic mice (n = 8) were placed beside the itch demonstrator mice (n = 8), and their behaviors were recorded.

To distinguish the effect of lidocaine alone, a lidocaine control group was also included. In this group, observer mice (n = 8) received only an intranasal lidocaine hydrochloride administration.

To eliminate the sense of hearing in the observer mice, kanamycin was intraperitoneally injected at a dose of 1000 mg/kg, followed by furosemide within 30 min. Confirmation of deafness was obtained through auditory brainstem response (ABR) testing within three days of the injection^15^. On the fourth day, the experiment was performed, and deaf observer mice (n = 8) were placed beside the itch demonstrator mice (n = 8).

#### Experience-dependent potentiation of contagious itch

We designed two arms to explore the influence of both previous and ongoing experiences of itch on contagious behavior in mice. Recognizing the significance of olfactory functions in contagious itch, we replicated the same experiments with anosmic mice.

##### Experiment 1: previous experience of itch

Eight observer mice, previously exposed to itch stimuli, were selected. Positioned alongside eight itch demonstrator mice, the observers received a pre-experimental injection of 100 µg/site of C48/80 one day before the designated experimental day.

##### Experiment 2: current (ongoing) experience of itch

This experiment aimed to determine whether contagious itch manifests as imitative behavior, facilitated by simultaneous injection of a pruritogen into the observer mouse while observing the demonstrator's behavior. Two groups of mice were involved: In the first group, eight observer mice received a sub-effective dose of C48/80 (30 µg/site) and were placed adjacent to itch demonstrator mice, which also received the same sub-effective dose of C48/80 (30 µg/site). In the second group, eight observer mice were injected with the sub-effective dose of C48/80 (30 µg/site) and were situated next to itch demonstrator mice receiving an effective dose of C48/80 (100 µg/site).

### Measurement of c-Fos expression in the brain

#### Western blot

The mouse brain regions were dissected by consulting an expert veterinary anatomist per previous descriptions (n = 4 in each group)^16,17^. To summarize, following euthanization, the head was detached from the remainder of the body and the muscles and bones surrounding the scalp were cut away. The olfactory bulb was removed with the tapered arrow-shaped spatula end. By using a razor blade, the posterior segment, along with its constituents (hippocampus, thalamus, and hypothalamus), was separated from the anterior segment. The next step was to remove the cortex followed by the separation of the hippocampus from its ventral and dorsal belongings. On the medial and inferior side of the hippocampus, the thalamus appears as an oval-shaped region enclosed by fornix. Upon locating this region, the thalamus and hypothalamus were separated for further experiments.

The obtained tissues for western blotting were then lysed using RIPA lysis buffer (50 mM Tris–HCl, 1% Triton X-100, 150 mM NaCl, 1 mM Ethylene glycol tetraacetic acid, 0.25% sodium deoxycholate, and 1 mM NaF) containing 1 mM Na_3_VO_4_, 1 mM phenylmethane sulfonyl fluoride or phenylmethylsulfonyl fluoride, and protease inhibitor. The lysates were separated by centrifugation at 14,000 rpm for 20 min at 4 °C. Protein concentration was determined using the Bradford Protein Quantification kit (Cat No: DB0017, DNAbioTech, Iran) following the manufacturer's instructions. Subsequently, the tissue lysates were combined with an equal volume of 2X Laemmli sample buffer. Lysates containing 20 μg of protein were then subjected to SDS-PAGE after a 5-min boiling step and transferred to a 0.2 μm immune-Blot™ polyvinylidene difluoride (PVDF) membrane (Cat No: 162-017777; Bio-Rad Laboratories, CA, USA).

For membrane blocking, 5% BSA (Cat No: A-7888; Sigma Aldrich, MO, USA) in 0.1% Tween 20 was applied for 1 h. Following blocking, membranes were incubated with Anti-c-Fos (Cat No: ab222699, Abcam) or anti-β actin-loading control antibodies (Cat No: ab8227, Abcam) for 1 h at room temperature. Subsequently, membranes were washed three times with TBST and incubated with goat anti-rabbit IgG H&L (HRP) (Cat No: ab6721; Abcam) secondary antibody. The membranes were then exposed to enhanced chemiluminescence (ECL) for 1–2 min. Protein expression was normalized to β-actin. Densitometry of protein bands was conducted using gel analyzer Version 2010a software (NIH, USA). Specifically, the percentage area under the curve of each band was divided by the percentage area under the curve of its corresponding actin band. The calculated values were then compared between groups, as previously described^18^.

### Statistical analysis

Data were analyzed using Student's *t*-test, one-way and two-way analysis of variance (ANOVA) with GraphPad Prism 8.0 software and Tukey’s multiple comparisons tests. Analysis with the *t*-test was used for some of the experiments. Data are expressed as mean ± standard error of the mean (SEM), and a *p*-value < 0.05 was considered statistically significant.

## Results

### Contagious itch happens in mice: the importance of social modulation and physical encounter with a cagemate

We tested the presence of contagious itch in mice while observing a mouse with excessive scratching behavior. The mice that received an intradermal injection of saline or C48/80 were used as a control or scratching demonstrator for observer mice that were its cagemate for at least two weeks^14^.

**Figure 1:**
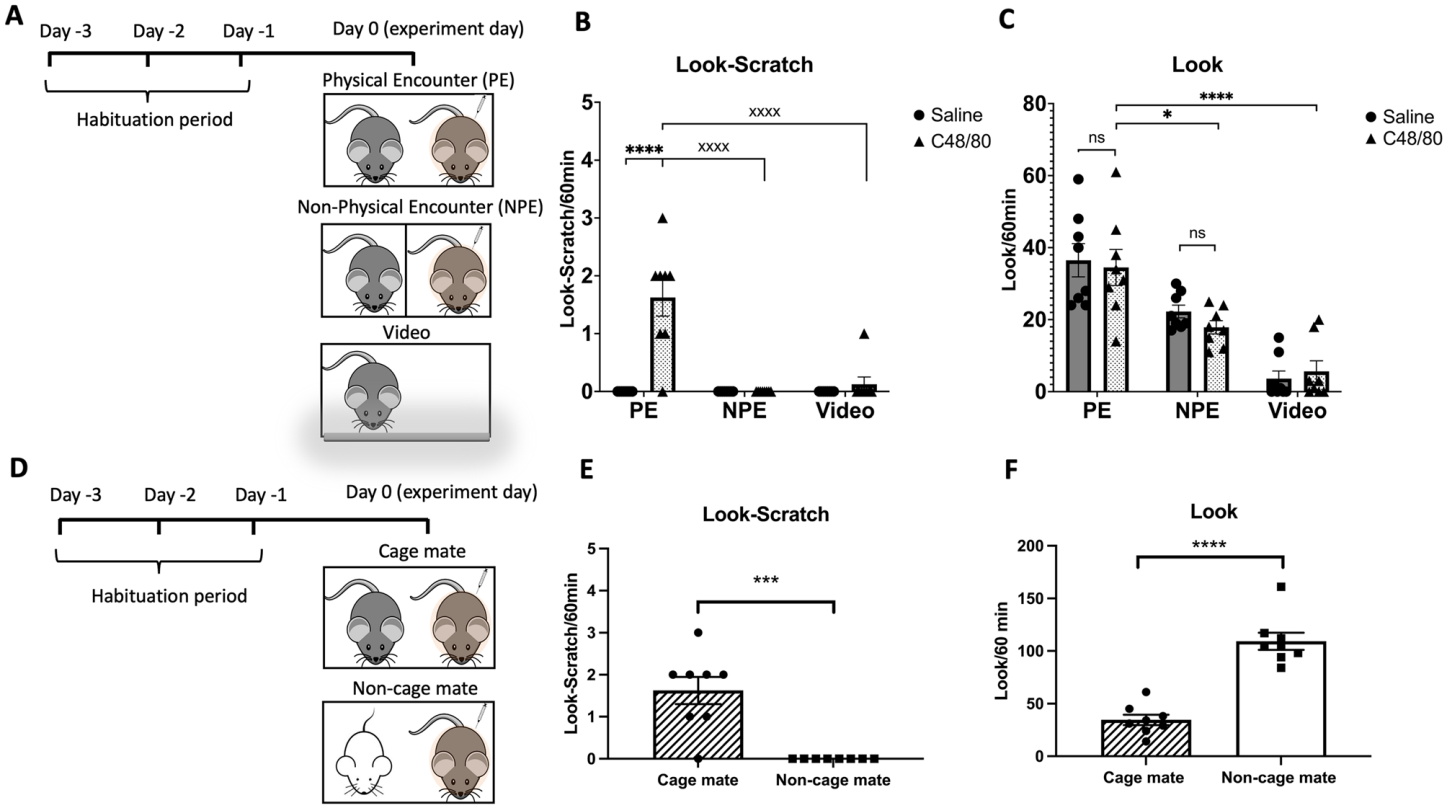
Contagious itch behavior in mice: the role of social modulation and physical encounter with a cagemate. (**A**) Cagemate mice were habituated for three days before the experiment. Physical Encounter (PE) involved direct contact, Non-Physical Encounter (NPE) utilized a transparent barrier, and the third condition exposed mice to a video of the scratching demonstrator. Bar graphs represent the number of **(B)** look-scratch and **(C)** looks exhibited by observer mice observing a scratching cagemate demonstrator that has been injected with the effective dose of C48/80 (100 µg/site) in the physical encounter (PE) and non-physical encounter (NPE) groups relative to their corresponding control (Ctrl) groups injected with normal saline. Significant differences are indicated by asterisks (^****^ *P* < 0.0001, ^xxxx^ *P* < 0.0001, and ^*^ *P* < 0.05 for comparisons with C48/80 (PE). **(D)** The familiar group of mice shared a cage for two weeks. In the unfamiliar group, mice from different family cages were habituated separately before the experiment. The mean number of **(E)** look-scratch and **(F)** looks in observer mice when placed next to a cagemate or non-cagemate (Correction in all similar positions: non-cagemate) scratching demonstrator. Values are presented as mean ± SEM.

To account for all possibilities, we divided the mice into two groups: physical encounter (PE) and non-physical encounter (NPE). In the PE group, both the observer and demonstrator were placed in the same box. In the NPE group, mice were separated by a thin, perforated, lucent plastic layer that allowed for the transmission of auditory, olfactory, and visual cues while keeping them physically apart **(**Fig. 1A). The total number of scratching bouts induced by the effective dose of C48/80 was comparable between the PE and NPE demonstrator mice (125.8 ± 9.5 vs. 136.8 ± 18.3, F (3, 28) = 39.77, *P* > 0.05) (Supplementary Fig. S4A). We also adopted the methodology described by Y.Q. Yu et al.^7^ to replicate the phenomenon of video-induced contagious itch in mice.

During the one-hour experiment, the PE group of mice with C48/80-injected demonstrator exhibited clear contagious itch behavior compared to the corresponding mice in NPE and video groups (Fig. 1B, ^XXXX^ pairwise comparison). In the PE pair, the observer mice with saline-injected demonstrator showed no coincidental look-scratch behavior vs. the one with C48/80-injected demonstrator (0.0 ± 0.0 vs. 1.6 ± 0.3 look-scratch, *P* < 0.0001) **(**Fig. 1B, ^****^ pairwise comparison). A two-way ANOVA showed the significant effect of physical encounter (F (2, 42) = 20.35, *P* < 0.0001), the effect of demonstrator itching behavior (F (1, 42) = 25.41, *P* < 0.0001), and the interaction of these two factors (F (2, 42) = 20.35, *P* < 0.0001) on look-scratch behavior.

The total number of looking behavior was similar between the control and scratching pairs in the PE group (34.5 ± 4.9 vs. 36.5 ± 4.6 looks, *P* > 0.05) **(**Fig. 1C), while the total number of looks towards the itch demonstrator in the PE group (1.9-fold increase) was significantly higher than that in the NPE (1.6-fold increase) (34.5 ± 4.9 compared to 17.8 ± 1.8 looks, *P* = 0.014) (Fig. 1C, ^*^ pairwise comparison) and video groups (34.5 ± 4.9 compared to 5.6 ± 2.9, *P* < 0.0001) (Fig. 1C, ^****^ pairwise comparison). A two-way ANOVA showed the significant effect of physical encounter (F (2, 42) = 43.45, *P* < 0.0001), but a non-significant effect of demonstrator itching behavior (F (1, 42) = 0.3, *P* = 0.59), and the interaction of these two factors (F (2, 42) = 0.47, *P* = 0.62) on the number of looks. The total number of scratching bouts between PE and NPE observer mice was comparable over the 60 min of the experiment (Supplementary Fig. S2A).

When a non-cagemate demonstrator mouse as an unfamiliar one was co-housed in the physical encounter condition (Fig. 1D), the observer mice did not exhibit any look-scratch behavior (0.0 ± 0.0 compared to 1.6 ± 0.3 look-scratch in the control group, *P* = 0.0002) (Fig. 1E). However, the total number of looks towards the non-cagemate itch demonstrator was increased (109.3 ± 8.2 compared to 34.5 ± 4.9 looks in the control group, *P* < 0.0001) (Fig. 1F). The total number of scratching bouts over 60 min was comparable between cagemate and non-cagemate observer mice (Supplementary Fig. S2B).

The decrease in contagious itch behavior in non-cagemate pairs is likely due to reduced empathy for scratching bouts of the unfamiliar mouse or stress to the unfamiliar mouse. Conversely, acute stress induced by foot shock in observer mice did not affect the number of look-scratch behavior (1.6 ± 0.3 vs. 1.8 ± 0.2, *P* > 0.05) (Supplementary Fig. S3A), looks (34.5 ± 4.9 vs. 29.3 ± 1.1, *P* > 0.05) (Supplementary Fig. S3B), or total scratching bouts (13.5 ± 3.4 vs. 10.7 ± 1.8, *P* > 0.05) (Supplementary Fig. S3C) in the physical encounter condition. This finding indicates that stress is irrelevant in producing contagious itch behavior in mice.

### The importance of olfactory signals in the communication of itch from one mouse to another

**Figure 2:**
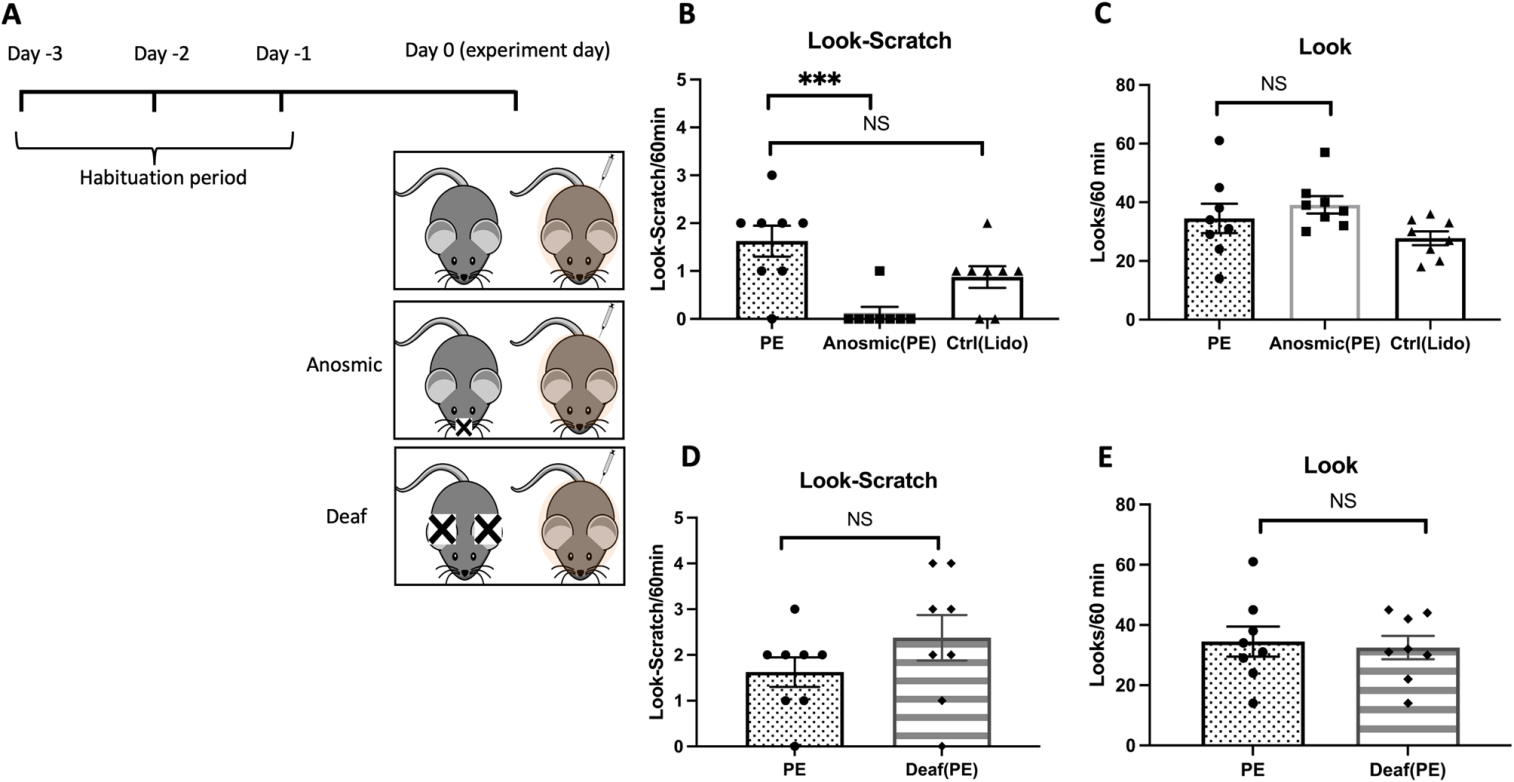
The importance of olfactory cues in itch communication between mice. **(A)** Anosmia in observer mice was induced by intranasal zinc sulfate post-lidocaine anesthesia for four days. Auditory impairment was achieved with kanamycin (1000 mg/kg) and furosemide. Anosmic (PE) animals exhibited significantly fewer contagious scratching behaviors (^***^ *P* < 0.001 compared to PE controls without anosmia) **(B)** but a similar number of looking behaviors **(C)** as the physical encounter (PE) group. The mean number of **(D)** look-scratch and **(E)** looks of deaf observer mice relative to the physical encounter (PE) group. Values are presented as mean ± SEM. NS: not significant.

We found that sensory cues from co-housed mice, rather than physically separated cagemates, play a role in contagious itch behaviors. This discovery has led us to explore the transmission of sensory modalities in contagious itch by selectively blocking different sensory inputs, such as rendering mice anosmic or deaf (Fig. 2A).

Anosmia was induced in mice by administering lidocaine and zinc sulfate (ZnSO_4_) intranasally four days before the test. We confirmed that the treated mice were anosmic by observing an increased time taken to search for food, as compared to the primary PE controls that did not have anosmia (203.9 ± 28.4 s vs. 17.2 ± 2.5 s, F (2, 21) = 43.25, *P* < 0.0001) (Supplementary Fig. S5A, ^****^ pairwise comparison). Anosmic animals exhibited significantly fewer contagious look-scratch behavior compared to non-anosmic mice (0.1 ± 0.1 vs. 1.6 ± 0.3 look-scratch, F (2, 21) = 9.81, *P* = 0.0007) (Fig. 2B, ^***^ pairwise comparison), even though they showed comparable looking behavior (39.1 ± 2.9 vs. 34.5 ± 4.9 looks, F (2, 21) = 2.50, *P* > 0.05) (Fig. 2C) and total scratching bouts (Supplementary Fig. S2C). Lidocaine alone did not significantly alter either smelling ability to find hidden food (17.2 ± 2.5 vs. 15.0 ± 1.3 time to find food, F (2, 21) = 43.25, *P* > 0.05) (Supplementary Fig. S5A) or contagious scratching behavior (0.8 ± 0.2 vs. 1.6 ± 0.3 look-scratch, F (2, 21) = 9.81, *P* > 0.05) (Fig. 2B).

To determine whether deafness can alter the contagious itch behaviors similar to anosmia, we assessed the behaviors of deaf observer mice. Deafness was induced by intraperitoneal injection of kanamycin and furosemide, given 30 min apart, three days before the experiment. The weight gain in mice due to kanamycin injection after three days was insignificant in this study (29.6 ± 0.5 vs. 28.3 ± 0.3, *P* > 0.05) (Supplementary Fig. S5B). We found that hearing loss and resultant lack of auditory cues did not significantly alter the look-scratch behaviors (2.3 ± 0.4 vs. 1.6 ± 0.3 look-scratch, P > 0.05) (Fig. 2D), looking behavior (32.5 ± 3.8 vs. 34.5 ± 4.9 looks, *P* > 0.05) (Fig. 2E), or the total scratching bouts (Supplementary Fig. S2D) in observer mice compared to healthy mice in the PE condition.

### The past and present encounter with itch stimuli modulates the contagious itch

**Figure 3:**
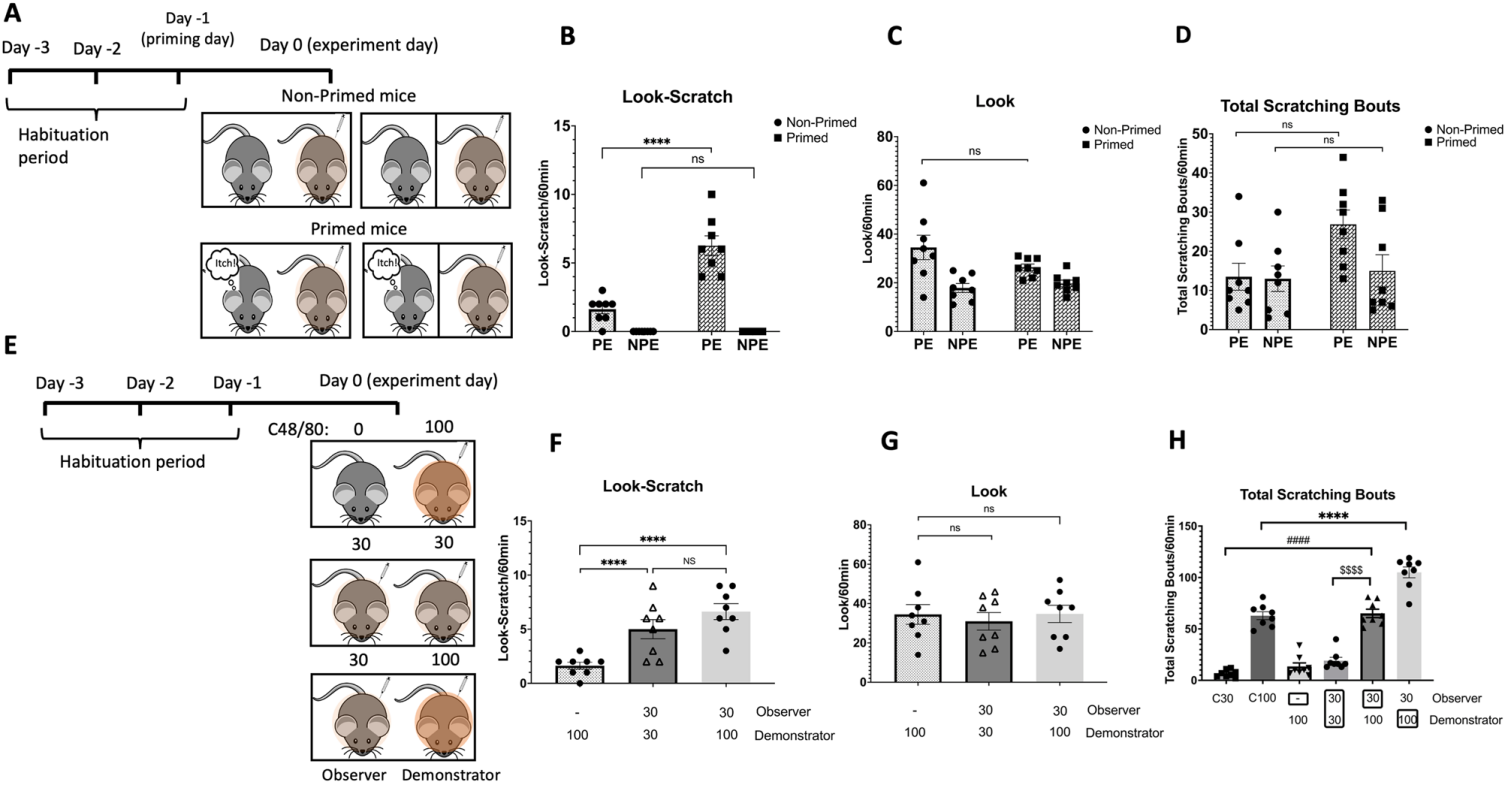
Prior itch experience modulates look-scratch behavior. (**A**) Primed observer mice, pre-exposed to itch stimuli, with 100 µg/site of C48/80 one day before the experiment. Representative bar graphs show the mean number of **(B)** look-scratch, **(C)** looks, and **(D)** total scratching bouts of primed observer mice in comparison to mice without prior itch experience in the physical encounter (PE) and non-physical encounter (NPE) conditions (^****^ *P* < 0.0001 shows the comparison between primed and non-primed mice in the PE condition). **E)** Group 1: Observers got no pruritogen, paired with demonstrators receiving an effective C48/80 dose (100 µg/site). Group 2: Both observers and itch demonstrators got a sub-effective C48/80 dose (30 µg/site). Group 3: Observers got the sub-effective dose, while adjacent itch demonstrators received the effective dose (100 µg/site) of C48/80. Representative bar graphs of the mean number of **(F)** look-scratch, **(G)** looks, and **(H)** total scratching bouts of observer mice in different groups. The observer mice injected with the pruritogen exhibited a significant increase in both look-scratch responses compared to the observer that received no pruritogen (^****^ *P* < 0.0001 and ^####^ *P* < 0.0001 pairwise comparisons). The total scratching bouts of pruritogen-injected mice were higher when paired with mice exhibiting scratching behavior (^$$$$^ *P* < 0.0001 compared C48/80 (30 µg/site) injected mice placed next to the demonstrator received 30 or 100 µg/site of C48/80, ^****^ *P* < 0.0001 and ^####^ *P* < 0.0001 compared C48/80-injected mice placed alone or next to a demonstrator with scratching activity. Values are presented as mean ± SEM. NS: not significant.

Individuals afflicted with pruritic skin disease exhibit heightened susceptibility to contagious itch cues compared to their healthy counterparts^19^. Therefore, we focused on assessing the impact of prior or current exposure to an acute and intense itch on the contagious itch behavior in mice (Fig. 3A,E). Our data showed the significant effect of previous itch priming (F (1, 28) = 33.86, *P* < 0.0001), physical encounter (F (1, 28) = 98.17, *P* < 0.0001), and the interaction of previous itch priming and physical encounter (F (1, 28) = 33.86, *P* < 0.0001) on contagious look-scratch behavior (Fig. 3B). One day after experiencing an itch induced by the effective dose of C48/80, primed observer mice displayed potentiated look-scratch behavior compared to those without prior exposure (6.2 ± 0.7 vs. 1.6 ± 0.3 look-scratch, *P* < 0.0001) (Fig. 3B, ^****^ pairwise comparison), despite similar numbers of looks between the two groups (26.3 ± 1.3 vs. 34.5 ± 4.9 looks, F (3, 28) = 7.09, *P* > 0.05) (Fig. 3C). However, physical contact was still necessary for contagious itch, as mice with prior itch experience did not exhibit any look-scratch behavior in non-physical encounters (Fig. 3B). The data analysis by two-way ANOVA demonstrates that the number of looks was affected by physical encounter (F (1, 28) = 16.93, *P* < 0.0001), but not by the previous itch experience (F (1, 28) = 1.223, *P* > 0.05, physical encounter × itch priming (F (1, 28) = 3.132, *P* > 0.05)) (Fig. 3C). Furthermore, the total number of scratching bouts was notably affected by prior exposure to acute itch (F (1, 28) = 4.465, *P* = 0.04), although post hoc analysis did not reveal any significant difference in total scratching bouts between the primed mice and the non-primed group (26.8 ± 3.6 vs. 13.5 ± 3.4, *P* = 0.03) (Fig. 3D).

In the second phase of this experiment, we investigated whether concurrent experience of itch affects contagious itch behavior. This exploration involved administering sub-effective doses of pruritogen (C48/80, 30 µg/site) to the observer mouse just before the experiment, ensuring minimal itch experience while simultaneously witnessing the scratching bouts of the demonstrator (Fig. 3E). We found a significant increase in look-scratch responses (5 ± 0.8 and 6.6 ± 0.7 vs. 1.6 ± 0.3, F (2, 21) = 13.70, *P* < 0.0001) (Fig. 3F, ^****^ and ^####^ pairwise comparisons) in 30 µg/site C48/80-injected observer mice placed next to the demonstrator that was injected with 30 or 100 µg/site of C48/80, compared to non-injected observer mice. The number of looks is comparable between the observers (31 ± 4.4 and 34.7 ± 4.3 vs. 34.5 ± 4.9, F (2, 21) = 0.2, *P* = 0.8) (Fig. 3G). Although there is no significant difference in the number of look-scratch bouts between the 30 µg/site of C48/80-injected observers next to the demonstrator mice injected with either 30 or 100 µg/site of C48/80 (5 ± 0.8 vs. 6.6 ± 0.7, F (2, 21) = 13.7, *P* > 0.05) (Fig. 3F), the total number of scratching bouts increased, indicating that the observer's understanding of itch was potentiated in the presence of the demonstrator mouse with a higher number of scratching bouts (19.2 ± 3.1 vs. 62.8 ± 3.8, F (5, 42) = 108.5, *P* < 0.0001) (Fig. 3H, ^$$$$^ pairwise comparison). Moreover, when mice injected with a pruritogen are placed together, they exhibit a higher level of scratching behavior than when they are alone and injected with the same pruritogen (Fig. 3H, ^****^, and ^####^ pairwise comparisons).

Taken together, our results suggest that the observer's familiarity not only influences contagious itch but also impacts overall scratching behavior. This supports the idea that contagious itch is a manifestation of empathic and imitative tendencies, underscoring the need for further investigation into both aspects.

### The influence of olfactory inputs on enhanced contagious itch, regulated by previous and present experiences of itch

**Figure 4:**
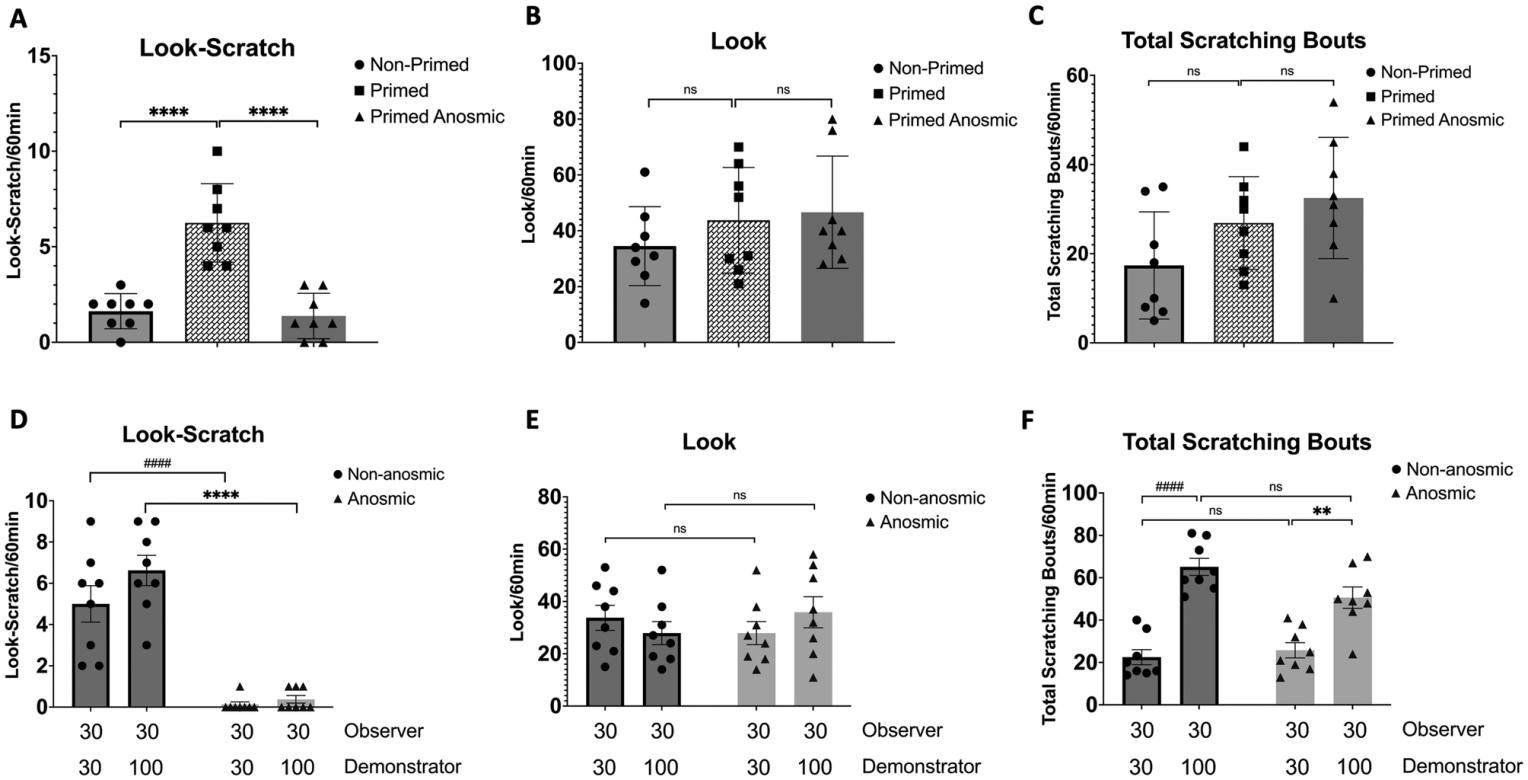
Olfactory inputs regulate enhanced contagious itch by past and current experiences of itch. (**A**) The primed anosmic mice exhibited a significant reduction in look-scratch behavior, an effect potentiated in non-anosmic primed mice (^****^ *P* < 0.0001 pairwise comparisons). The number of looks **(B)** and total scratching bouts **(C)** are comparable between the groups. Itch priming was induced by the injection of 100 µg/site of C48/80 one day before the designated experimental day. **(D)** Anosmic mice with concurrent experience of itch induced by the sub-effective dose of C48/80 exhibited significantly lower counts of look-scratch responses but a comparable number of **(E)** looks and **(F)** total scratching bouts compared to their non-anosmic counterparts. Values are presented as mean ± SEM.

Considering the essential role of olfactory cues on contagious itch, we primed anosmic mice with one event of acute itch a day before exposing them to demonstrator mice. Surprisingly, we found a significant decrease in look-scratch behavior in anosmic primed mice compared with a non-anosmic primed group (1.3 ± 0.4 vs. 6.2 ± 0.7, F (2, 21) = 28.05, *P* < 0.0001) (Fig. 4A, ^****^ pairwise comparisons), while the number of looks (46.6 ± 7.1 vs. 43.7 ± 6.6, *P* > 0.05) (Fig. 4B) and total scratching bouts (32.5 ± 4.8 vs. 26.8 ± 3.6, *P* > 0.05) (Fig. 4C) are comparable between the groups.

We then repeated the experiment where anosmic observer mice were exposed to a sub-effective dose of the pruritogen (C48/80, 30 µg/site) alongside a demonstrator injected with either 30 or 100 µg/site of C48/80. The results indicated a significant impact of the olfactory system on the frequency of look-scratch behaviors (F (1, 28) = 90.45, *P* < 0.0001). However, neither the demonstrator's scratching (F (1, 28) = 2.56, *P* = 0.12) nor the interplay between olfaction and the demonstrator’s scratching bouts (F (1, 28) = 1.38, *P* = 0.25) significantly influenced contagious itch. Anosmic mice exhibited significantly lower counts of look-scratch responses compared to non-anosmic counterparts (0.1 ± 0.1 vs. 5 ± 0.8, *P* < 0.0001 and 0.3 ± 0.1 vs. 6.6 ± 0.7, *P* < 0.0001) (Fig. 4D, ^####^, and ^****^ pairwise comparisons) despite displaying a comparable number of looks (27.8 ± 4.4 vs. 33.7 ± 4.8, *P* > 0.05, and 35.8 ± 6 vs. 31.8 ± 5.1, *P* > 0.05) (Fig. 4E) and total scratching bouts (20.7 ± 3.6 vs. 22.5 ± 3.5, *P* > 0.05, and 50.6 ± 5 vs. 65.1 ± 4, *P* > 0.05) (Fig. 4F).

### Brain regions involved in contagious itch behavior

**Figure 5:**
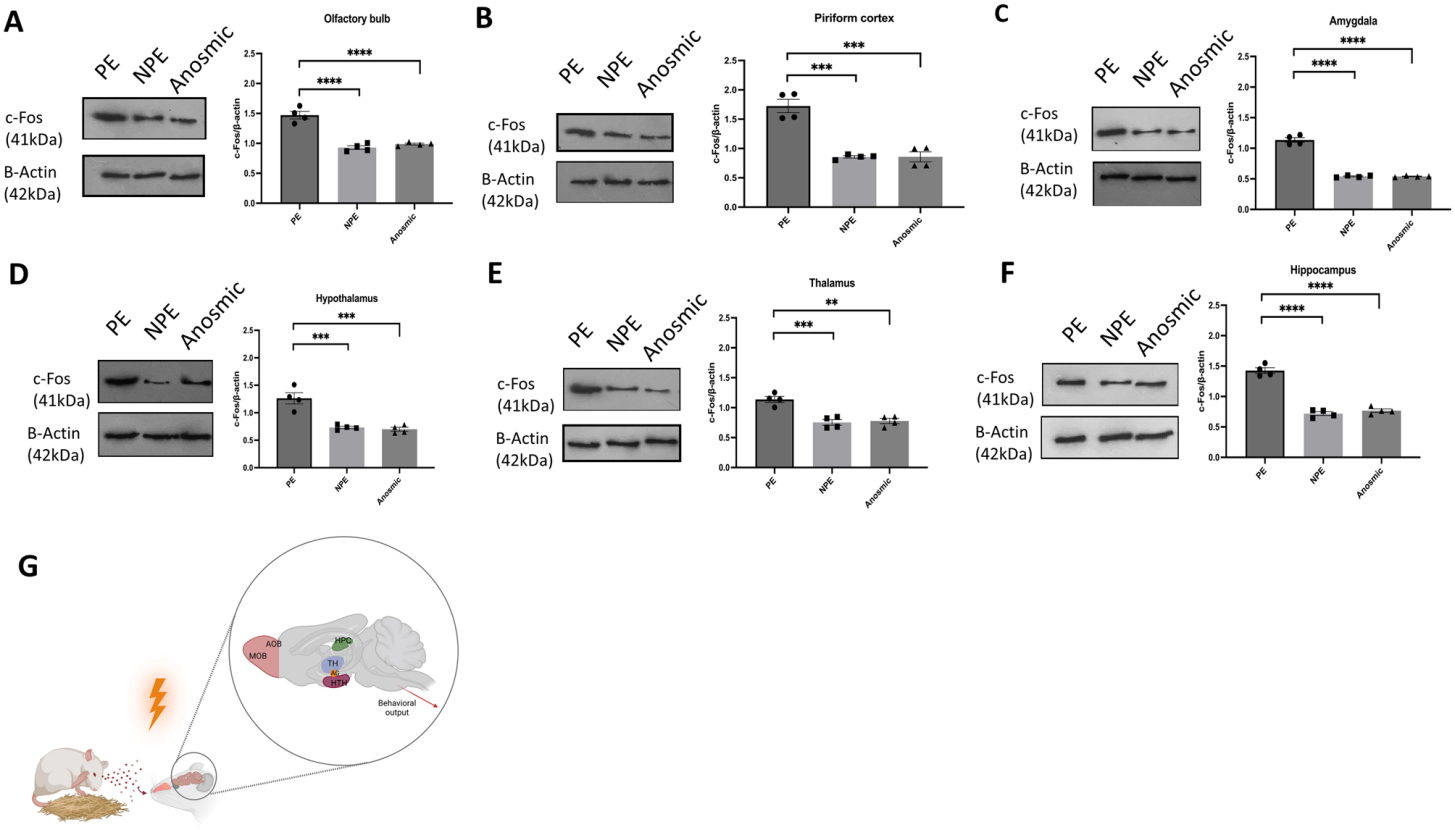
Activated brain regions in contagious scratching behavior. (**A**–**F**) Western blot depicting c-Fos protein expression in various brain regions of observer mice after physical (PE) and non-physical encounters (NPE) with scratching demonstrators, as well as anosmic observers exposed to adjacent itch demonstrators (n = 4).** (G)** Illustration representing the findings of our experiment. The contagious itch can be seen in mice models of the same sex, age, strain, and home cage while the olfactory system is intact, and the physical encounter is not restrained. This behavior is strengthened in mice with previous itch exposure and originates from empathetic behaviors. The olfactory bulb (containing the main olfactory bulb (MOB) and the accessory olfactory bulb (AOB)), hippocampus (HPC), thalamus (TH), amygdala (AG), and hypothalamus (HTH) are involved in the signal transmission of contagious itch behavior. Created with BioRender.com.

To identify the brain regions involved in contagious itch behaviors, we measured the c-Fos protein by western blot in various brain regions of PE, NPE and anosmic mice witnessing scratching demonstrator following a one-hour video recording of contagious itch behaviors. Our data showed a significant increase in c-Fos expression in several brain regions of mice with contagious itch behavior (PE) relative to the mice without contagious itch (NPE), including the olfactory bulb (*P* < 0.0001), the piriform cortex (*P* = 0.0001), the amygdala (*P* < 0.0001) the hypothalamus (*P* = 0.0006), the thalamus (*P* = 0.0007), and the hippocampus (*P* < 0.0001) (Fig. 5A–G). Furthermore, comparing the anosmic observer alongside the scratching demonstrator compared to the non-anosmic observers, the c-Fos expression is significantly reduced in the olfactory bulb (*P* < 0.0001), the piriform cortex (*P* = 0.0001), the amygdala (*P* < 0.0001), the hypothalamus (*P* = 0.0004), the thalamus (*P* = 0.0011), and the hippocampus (*P* < 0.0001) of anosmic observer mice (Fig. 5A–G). See the original membrane image in Supplementary Fig. S6.

## Discussion

We observed contagious itch behavior in cagemate (corrected version in all similar positions: cagemate) mice through physical encounters and discovered that familiar olfactory cues are involved in the process of this behavior. In addition, we observed an increase in contagious itch behavior in mice previously exposed to itch. Our data suggest that observer mice develop representations through experience that enable them to understand others, reflecting empathy that is enhanced by familiarity and similarity to others' circumstances. Our findings are supported by c-Fos expression in brain regions linked to visual cues such as the SCN and the olfactory processing, including the olfactory bulb and the piriform. Our data also demonstrated the involvement of brain regions associated with affective and motor-motivational processes in mice that exhibited contagious itch, including the thalamus, the hypothalamus, the amygdala, and the hippocampus.

Several studies have investigated itch contagion in humans^3,6, 20–23^. Yu et al.^7^ and R. Gonzales-Rojas et al.^8^ reported that *C57BL/6 J* mice exhibit imitative scratching behavior. This occurs upon exposure to scratching videos or genetically modified mice with excessive scratching, highlighting the potential of visual cues in inducing contagious itch behavior in mice. In *C57BL/6 J* mice, observing conspecific scratching video induces scratching behaviors^7,8^. We examined whether observing scratching video similarly induces scratching behaviors in NMRI mice. However, video observing alone did not evoke contagious itch behaviors in NMRI mice. Instead, we successfully induced contagious itch in NMRI mice through frequent scratching bouts of physically contactable and familiar demonstrator mice. Similar to our results Liljencrantz et al.^24^ reported that contagious itch cannot be observed in C57BL/6 mice through observation of video demonstrating scratching bouts of histamine-injected mice, although the histamine model may have limitations due to the inadequate frequency and duration of scratching bouts required for contagious itch tests^25^. In our video demonstration experiment, the observer NMRI mouse showed less attention to the video as low as ca. 5 looks over a 60-min period. Instead of less frequent scratching bouts of demonstrator mice, fewer looks toward the video demonstration would result in no video-induced contagious itch behaviors in NMRI mice. Furthermore, JS Lu et al.^19^ used a four-iPad paradigm to test the effect of watching itching videos on mice, finding no significant itch-like responses but observing altered activities in the open field with mirrors.

We further found that placing a physical barrier against the demonstrator mouse likely leading to a reduced attention and, as a result, eliminated the contagious itch response. Additionally, we observed that the number of looks towards the demonstrator mouse reduced by 1.6–1.9-fold in the non-physical contact group compared to the physical contact group. This decreased attention toward the demonstrator mice may have contributed to the reduced contagious itch response in the non-physical contact group.

The present study revealed an intriguing finding that olfactory cues could contribute to the occurrence of contagious itch in NMRI mice. We noted that anosmic mice did not display any contagious itch behavior. Moreover, the potentiated contagious itch resulting from ongoing or prior experiences of itch diminished in anosmic observer mice. In line with these findings, the activation of the olfactory bulb and the piriform cortex was consistently observed during contagious itch behavior in mice. Olfaction is a crucial sensory modality in rodents, vital for regulating social behaviors and emotions^26^. Studies have identified the activation of the piriform cortex and olfactory bulb brain regions in animals displaying social behavior^27,28^, and we observed the same activation during the contagious itch setting, which is diminished in anosmic mice.

In rodents, olfactory information from the main and accessory olfactory bulb is sent directly to the amygdala, which further processes and relays it to the hypothalamus, regulating emotions and social behavior during observational fear learning^29^. Our data showed that the amygdala and the hypothalamus activation is abrogated in anosmic mice witnessing scratching demonstrator suggesting the connection of the olfactory system with the amygdala and the hypothalamus during contagious itch in mice. The olfactory-amygdala-hypothalamus pathway may also play a role in itch transmission in humans^30^. It is noteworthy that ZnSO_4_, used to induce anosmia in mice, may synergistically reduce itch signals by modulating transient receptor potential A1 (TRPA1), G-protein coupled receptor (GPCR)-39, and mas-related G-protein coupled receptor (Mrgpr)^31,32^.

There is a lack of consensus regarding whether contagious itch qualifies as a type of empathy^6,20, 21^. Empathy has been crucial for the survival of species throughout evolutionary history. Typically, empathy consists of emotional and cognitive processing, which involve experiencing and comprehending another's emotions, respectively. However, the cognitive component favors cognition over emotions, which excludes children and most non-human species, such as rodents, from experiencing empathy for others. Recent theories emphasize affective and cognitive empathy, highlighting shared emotional representations^33^.

It is clear that mice can share the emotional state of another (state matching) and provide comfort to a distressed party (consolation)^33,34^. Both of these behaviors rely on emotional contagion, which is the basic form of empathy that Elaine Hatfield first defined as emotional state matching between an observer and a target^33^. The co-occurrence of itch behaviors in familiar individuals also provides strong evidence for contagious itch as a form of empathy in mice, which is a compelling analog to empathy in humans.

Similar to empathic pain demonstrated by Langford, Dale et al.^35^, the empathic itch is dependent on familiarity between the observer and the suffering target. As previously reported for contagious pain in mice^35^ and humans^36^, the induction of a glucocorticoid stress response in the presence of a stranger may be the reason for blocking contagious itch. Recent evidence has also revealed that contagious itch can encode stressful information^9^. However, our results showed no significant changes in contagious behavior following acute shock stress, a model known to elevate glucocorticoid levels in serum and brain^11,37^. This finding excludes the possibility of a link between the activation of the hypothalamic–pituitary–adrenal (HPA) axis, which could have been triggered by the presence of a stranger, and the suppression of contagious itch in our model. Nevertheless, further experiments are needed to unravel the factors contributing to the reduction of contagious itch in strangers.

Furthermore, the data shows that there is no association between the memory of visual and olfactory information and the pain caused by the foot shock. Therefore, future studies should aim to fully define the relationship between familiarity and contagious itch and investigate whether this effect is related to corticosteroid levels.

Together, our results indicate that high attention to the demonstrator mouse involves physical contact, familiarity, and olfactory cues and that empathy is required for contagious itch induction. We discovered that itch experience enhances contagious itch, although it is not a requirement. In humans, previous itch experience in atopic dermatitis patients also amplifies the subjective sensory experience of contagious itch elicited by visual cues^3^. This data supports the perception–action model or perception/action model of empathy for the contagious itch, which states that observers understand others' emotions through experience and familiarity, explaining the association of empathy with similarity to and understanding of the other person's feelings^38^. Further research is required to determine if cognitive empathy is present in rodents.

There are some limitations to our study. Firstly, we used male NMRI mice, which are less common in itch studies than C57BL/6 mice. Secondly, the connection between olfaction and vision would be valuable to be addressed, as there is strong evidence about the specific role of the visual pathway in contagious scratching^7,8^. Thirdly, since rodents use ultrasonic vocalization to convey social information, future studies should investigate whether this communication plays a role in contagious scratching. Moreover, we investigated the role of the hippocampus in the process of contagious itch, which may demonstrate the potential significance of memory in the manifestation of contagious itch, since the hippocampus and its surrounding cortex are crucial for encoding and retrieving memories^39^. Nonetheless, it should be noted that this finding could be obscured by the fact that continuous exposure to itch mediators could result in sensitization of itch pathways^40^. To shed light on this issue, further studies are necessitated to compare memory dependency with sensitization of itch pathways by intervening memory.

To summarize, we found that olfactory cues of familiar cagemates and physical contact are required for contagious itch behavior in mice, reflecting empathy that is enhanced by familiarity and similarity to others' circumstances. Studying empathic contagious itch in laboratory animals can help understand empathy's mechanisms, which arise from shared bottom-up processes across species. However, species-specific and age-dependent differences may exist in olfactory or visual cues that could create a link to memory-associated contagious itch behaviors. Comparing affective empathy in mice and humans is challenging due to the complexity of human empathic behavior. While contagious itch in mice suggests emotional contagion, it lacks the complexity of human empathy. Nonetheless, cognitive forms of empathy may be present during contagious itch in mice, suggesting a possible affective empathy component that requires further investigation.

### Supplementary Information

Supplementary Information 1.

Supplementary Video 1.

## Data Availability

All data generated or analyzed during this study are included in this article. Further inquiries can be directed to the corresponding author.
