# Supplementary material for: Social interactions and olfactory cues are required for contagious itch in mice
Source: Sci Rep. 2024 May 17;14:11334. doi: 10.1038/s41598-024-61078-3 (PMC11101621; doi:10.1038/s41598-024-61078-3)
Supplement: Supplementary file 1 — Supplementary Information 1. [file 41598_2024_61078_MOESM1_ESM.docx]

**Supplementary Information**

**Unraveling the Itchy Secrets: Social Interactions and Olfactory Cues Shape Contagious Itch in Mice**

**Maryam Shayan, Nazgol-Sadat Haddadi, Maryam Shokrian Zeini, Mohadese Shokrian Zeini, Hasti Tashak Golroudbari, Arya Afrooghe,** **Elham Ahmadi, Asma Rashki, Ahmad-Reza Dehpour**

This file contains the following:

Supplementary Figures 1 to 6.


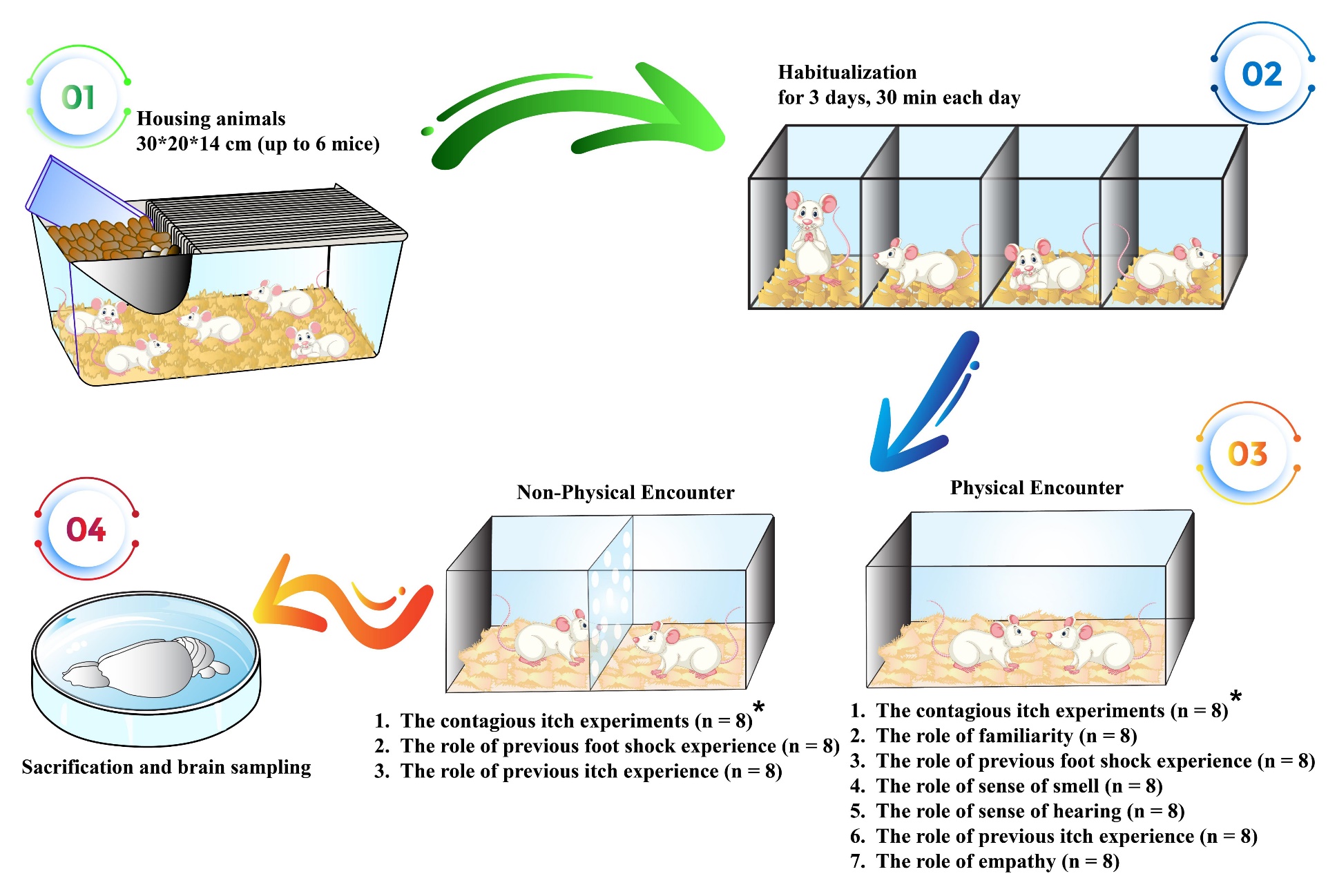


**Supplementary Figure S1. Experimental design of the study. 1)** Animals were housed in cages measuring 30 × 20 × 14 cm. The illustration depicts mice housed in groups of up to six in each cage. **2)** Each mouse was placed in a separate acrylic box (10 × 10 × 13 cm) with a small amount of bedding for 30 minutes daily for three days prior to the behavioral experiment in the habituation phase. **3)** The experimental groups for both the physical encounter and non-physical encounter experiments, along with the number of mice in each group, are displayed. ^*^Each observer mouse was paired with an individualized demonstrator, resulting in a total of 8 observer-demonstrator pairs in each group. **4)** After each experiment, the mice were sacrificed for brain sample collection in order to evaluate c-Fos expression in the brain. Created with Adobe Illustrator version v. 25.0.1

**
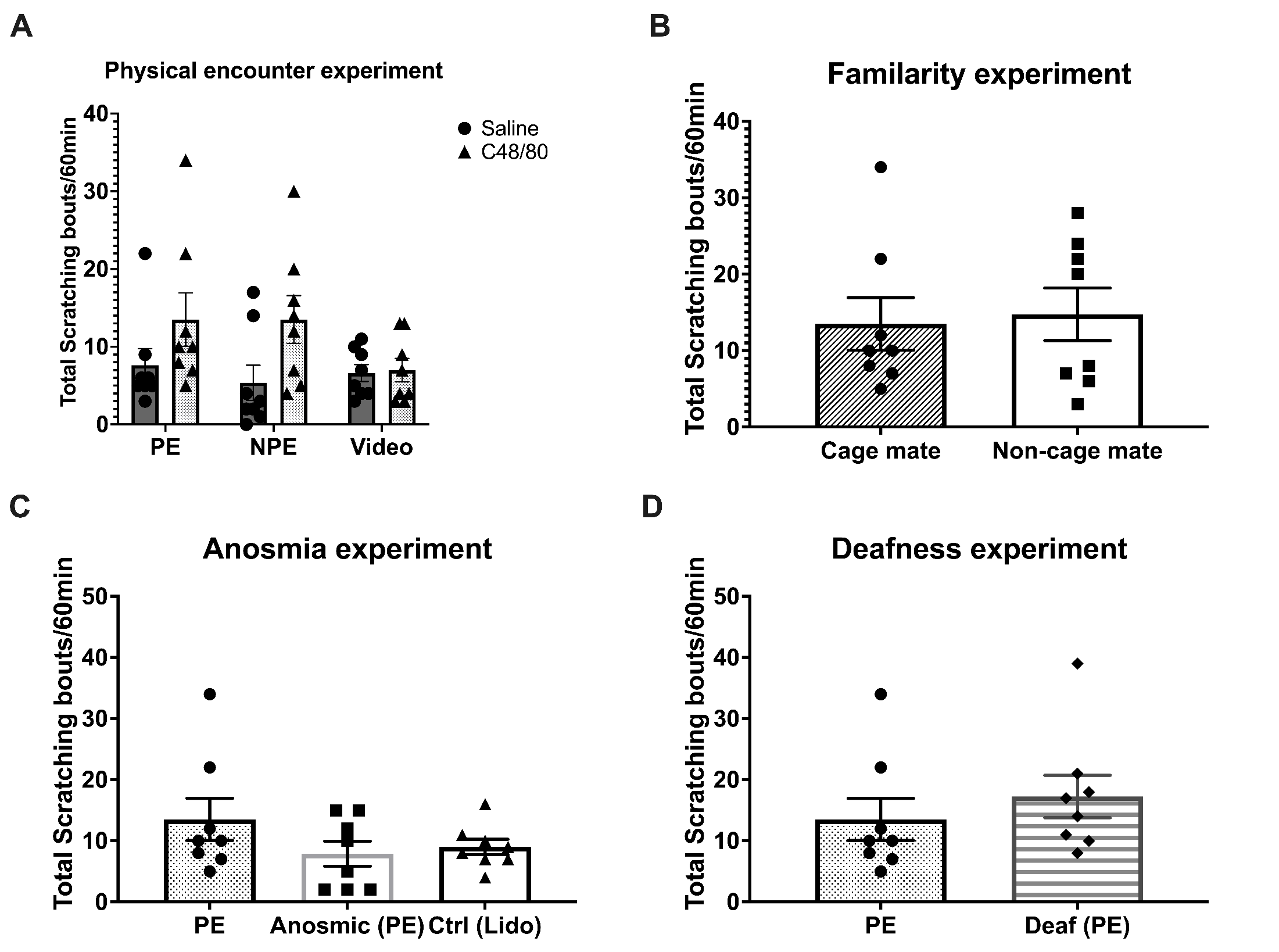
**

**Supplementary Figure S2. The total scratching bouts of observer mice in different experimental models used in the present investigation.** Bar graphs represent the mean number of total scratching bouts in observer mice in the **(A)** Primary models with the physical encounter (PE) and non-physical encounter (NPE), **(B)** Non-cagemate (familiarity) experiment, **(C)** Anosmia, and **(D)** Deafness experiments. Data are displayed as mean ± SEM of eight mice per group.

**
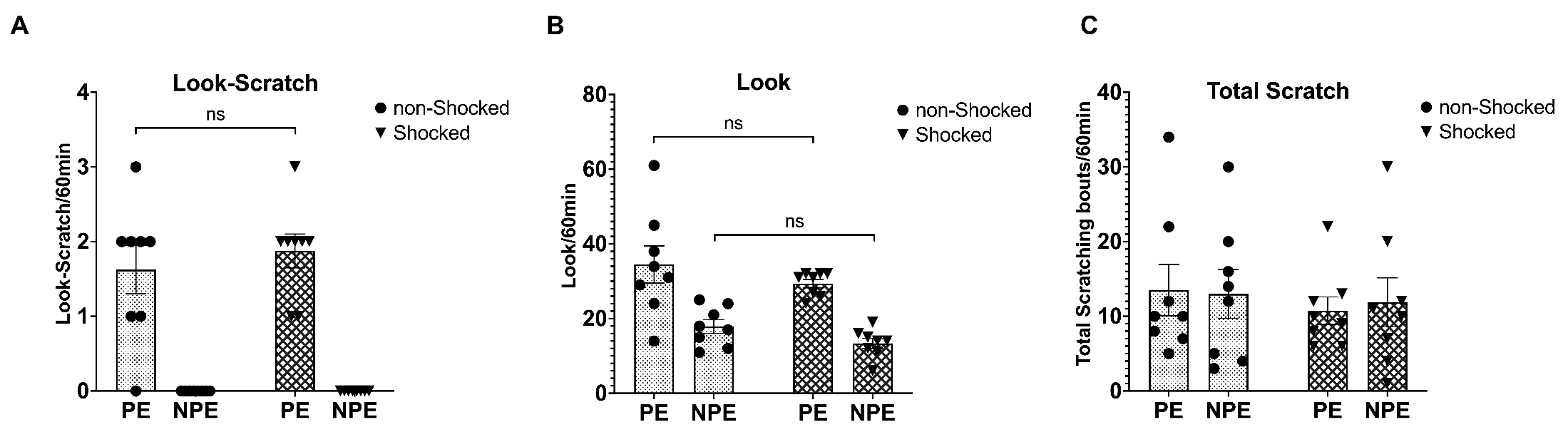
**

**Supplementary Figure S3. The effect of acute stress (shock) on contagious itch behavior.** The mean number of **(A)** look-scratch, **(B)** looks, and **(C)** total scratching bouts of observer mice under foot-shock-induced acute stress prior to the itch experiment in either physical encounter (PE) and non-physical encounter (NPE) condition when exposed to the scratching demonstrator. Data are presented as mean ± SEM of eight mice in each group. NS indicates non-significant differences.

**
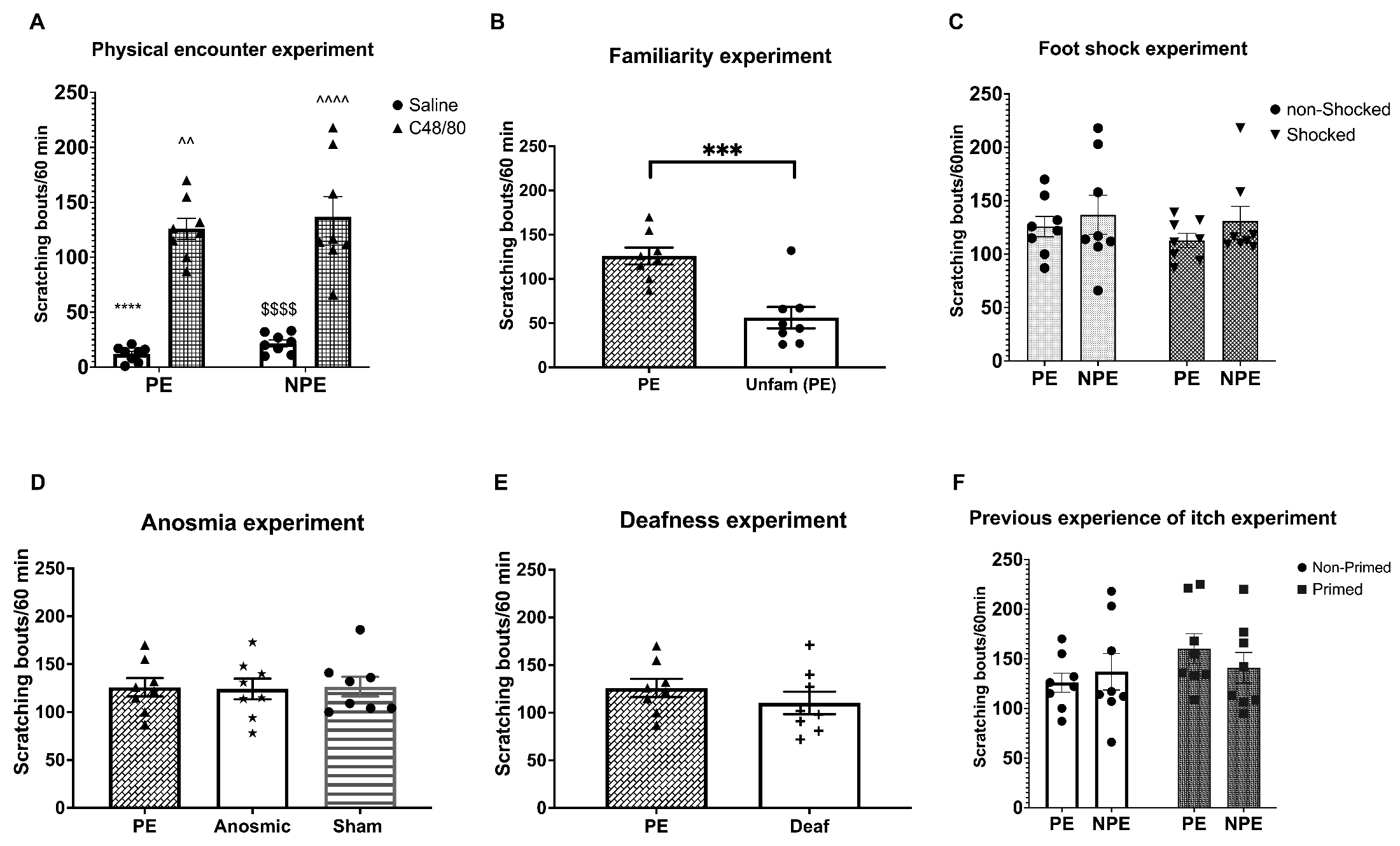
**

**Supplementary Figure S4. The demonstrator scratching bouts in different experimental models used in the present investigation.** Bar graphs represent the mean number of total scratching bouts in mice used as a scratching demonstrator in the **(A)** Primary models with the physical encounter (PE) and non-physical encounter (NPE) (^^^^ P < 0.01 and ^^^^^^ P < 0.0001 compared to the C48/80 (100). ^****^ P < 0.0001 compared to PE. ^$$$$^ P < 0.0001 compared to NPE), **(B)** Non-cage mate (familiarity) model, **(C)** Foot-shock-induced acute stress model, **(D)** Anosmia model, **(E)** Deafness model, and **(F)** Previous experience of itch model. Data are displayed as mean ± SEM of eight mice per group.

**
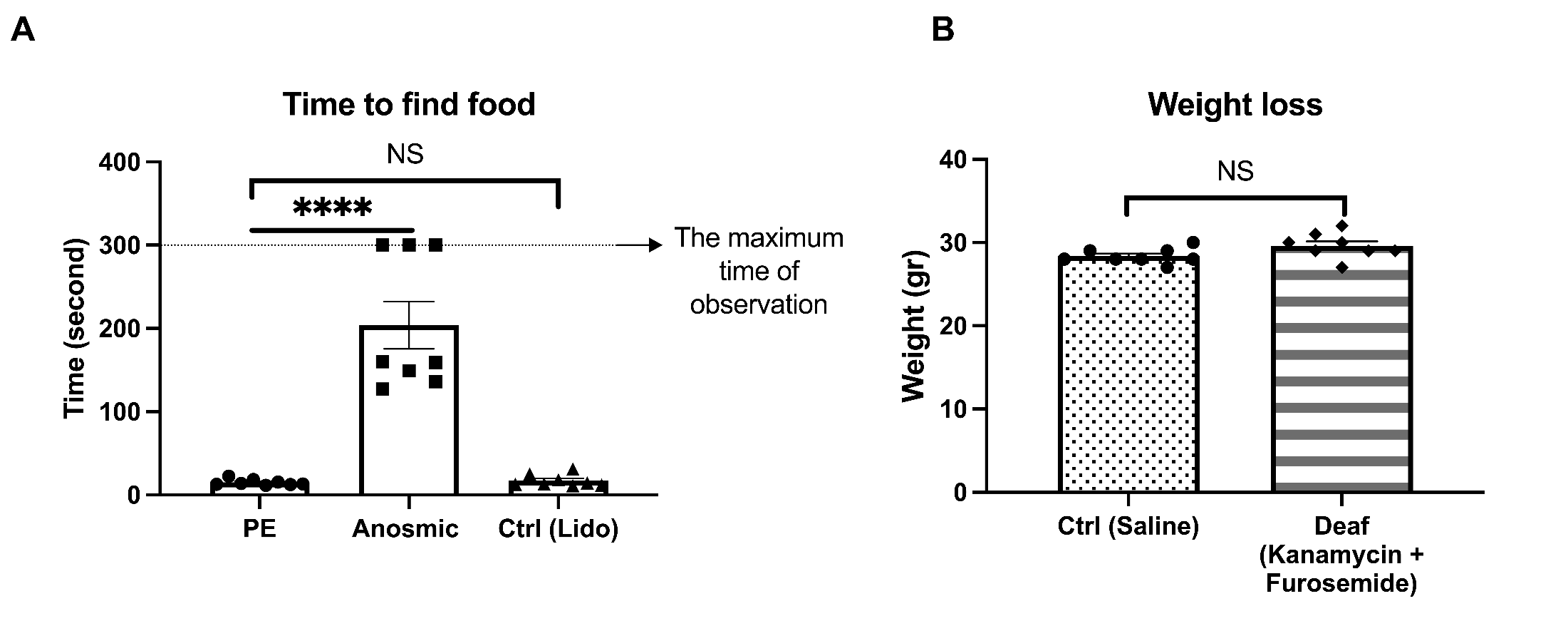
**

**Supplementary Figure S5. Implementation of anosmia and deafness in mice. (A)** Shows the time (in seconds) required to locate a buried chew. Anosmia was induced by intranasal administration of lidocaine and zinc sulfate (ZnSO_4_) for four days, confirmed by increased food search time. Lidocaine (Ctrl-lido) did not affect the sense of smell (^****^ P < 0.0001 compared to PE controls without anosmia). **(B)** Represents the changes in body weight of mice treated with an intraperitoneal injection of kanamycin and furosemide to induce deafness. Data are displayed as mean ± SEM of eight mice per group. NS indicates non-significant differences.

**
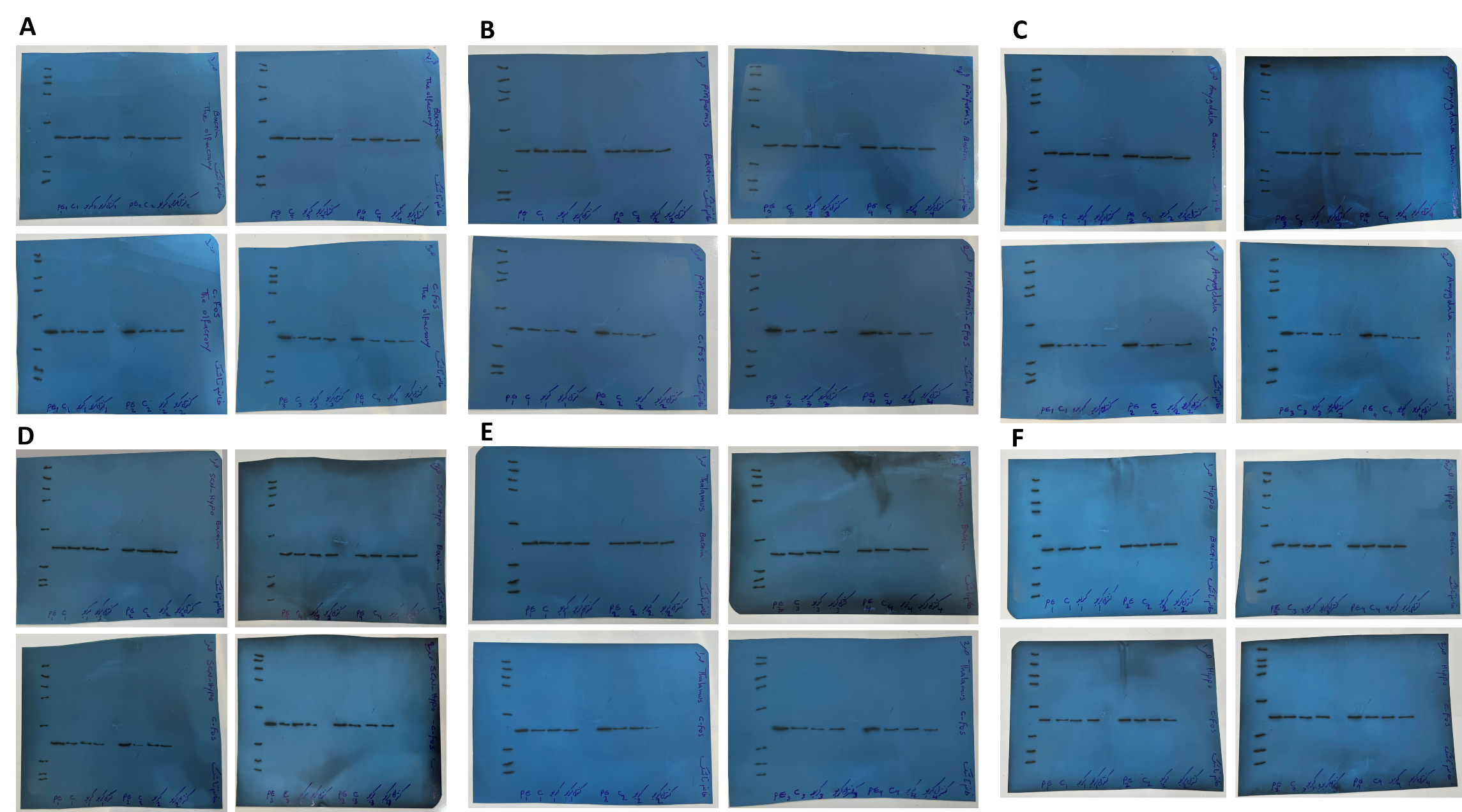
**

**Supplementary Figure S6. Original band images of western blot analyses.** Analysis of c-Fos protein expression in brain regions of physical encounter (PE), non-physical encounter (NPE), and anosmic mice observing scratching demonstrator after a one-hour video recording of contagious itch behaviors (n = 4). Original band images of the **(A)** olfactory bulb, **(B)** piriform cortex, **(C)** amygdala, **(D)** hypothalamus, **(E)** thalamus, and **(F)** hippocampus.
